# Role of mesenchymal stem cells, their derived factors, and extracellular vesicles in liver failure

**DOI:** 10.1186/s13287-017-0576-4

**Published:** 2017-06-06

**Authors:** Jie Wang, Panpan Cen, Jiajia Chen, Linxiao Fan, Jun Li, Hongcui Cao, Lanjuan Li

**Affiliations:** State Key Laboratory for Diagnosis and Treatment of Infectious Diseases, Collaborative Innovation Center for Diagnosis and Treatment of Infectious Diseases, the First Affiliated Hospital, Zhejiang University, 79 Qingchun Road, Hangzhou, 310006 China

**Keywords:** Mesenchymal stem cell, Liver failure, Exosome, Extracellular vesicles, Liver regeneration

## Abstract

Liver failure is a severe clinical syndrome with a poor prognosis. Mesenchymal stem cell (MSC) transplantation has emerged as a new intervention in treating liver failure. It is conventionally recognized that MSCs exert their therapeutic effect mainly through transdifferentiation. Recently, published articles have shown that MSCs work in liver failure by secreting trophic and immunomodulatory factors as well as extracellular vesicles (EVs) before transdifferentiation. In particular,MSC-derived EVs have shown similar curative effects as MSCs. Here we review the role of MSCs as well as their derived factors and EVs in liver failure and discuss the use of MSC-derived EVs instead of intact MSCs in treating liver failure.

**Electronic supplementary material:**

The online version of this article (doi:10.1186/s13287-017-0576-4) contains supplementary material, which is available to authorized users.

## Background

The liver is a vital organ that possesses a powerful regeneration capability and exerts detoxification functions in the body. However, when the outsource injury exceeds the compensatory ability, liver failure is induced. Liver failure, which can be caused by a variety of factors, is the inability of the liver to perform its normal synthetic and metabolic function as part of its normal physiology. It is a clinical syndrome that mainly manifests as coagulation dysfunction, jaundice, ascites, and hepatic encephalopathy. The overall prognosis of liver failure is poor due to its rapid progression, treatment difficulty, and the high medical costs associated with treatment. Currently, the ultimate therapeutic option is orthotopic liver transplantation, which is limited by organ shortage, high expense, and the requirement of lifelong immunosuppressive medication. Therefore, there is an urgent need for novel effective treatments.

Stem cell therapy has gained attention as a potential therapeutic approach for several diseases, including liver failure [1, 2]. Amongst several types of stem cells, mesenchymal stem cells (MSCs) are the most commonly used cells because they are easily acquired and free from ethical concerns. MSCs, a population of multi-lineage progenitor cells with self-renewal ability, are distributed widely throughout the body and can be isolated and purified from almost all tissues, such as bone marrow, umbilical cord, menstrual fluid, placenta, adipose tissue, dental pulp, and amniotic fluid. They have pleiotropic properties, including multiple differentiation, anti-apoptosis, angiogenesis, tissue repair promotion, anti-inflammatory activity, growth factor production, immunosuppression, and nerve protection properties [3].

MSCs have been used to effectively treat liver failure in both animal models [4–7] and clinical trials [2, 8, 9]. However, the exact mechanism of MSCs in liver regeneration remains unclear. In this work, we review the role of MSCs, their derived factors, and extracellular vesicles (EVs) in liver failure and attempt to identify the exact mechanism and specific component of MSC transplantation. Our goal of this review is to potentially describe any components that could serve as an alternative to MSCs.

### Transdifferentiation of MSCs into hepatocytes

It is conventionally recognized that MSCs exert their biological functions and play a role in treatment primarily by migrating to the damaged tissue [10], proliferating, transdifferentiating into hepatocytes, and replacing damaged cells [1]. MSCs obtained from various sources have been demonstrated to possess endodermal differentiation potential, which allows them to differentiate into functional hepatocyte-like cells in vitro under appropriate culture conditions [7, 11].

We developed an acute liver failure pig model induced by D-galactosamine (D-gal). We confirmed that human bone marrow-derived MSCs (BMSCs) are capable of transdifferentiating into hepatocytes and repopulating the liver in vivo through albumin secretion and the gene expression profile of transplanted human BMSC-derived hepatocytes. The concentration of human albumin in the animals increased to a stable concentration at 10 weeks and subsequently decreased at 15 weeks. The results of real-time quantitative polymerase chain reaction of human hepatocyte-specific genes and immunohistochemistry showed a similar trend. Immunohistochemistry revealed that human BMSC-derived hepatocytes were widely distributed in the hepatic lobule and accounted for approximately 30% of cells at 10 weeks. Immediate intraportal administration of human BMSCs could rescue liver failure in pigs effectively while peripheral vein transfusion failed to do so [4].

Studies have reported that MSC transplantation does not trigger a host-versus-graft response because MSCs lack co-stimulatory molecules and human leukocyte antigen class II [12]. Therefore, MSCs are suitable for autologous and allogeneic transplantation. Both animal experiments and clinical trials have revealed good tolerance of MSCs. Our data show that the number of hepatocyte-like cells decreased from weeks 15 to 20. This time period roughly corresponds to the normal life span of hepatocytes, which is 5 months. We infer that the MSCs did not trigger immune rejection or triggered a minimal immune rejection because of their low immunogenicity. MSC-derived hepatocytes may have naturally expired and it is unclear whether they induce immune rejection. However, the liver was shown to recover from acute liver failure regardless of whether these cells induced immune rejection or not. It may be beneficial for the body to naturally eliminate MSCs.

Similar animal experiments have certified the transdifferentiation and therapeutic ability of MSCs from other sources. Human placental MSCs extended the survival time of pigs in an acute liver failure model induced by D-gal. Human hepatocyte-specific markers were detected using immunohistochemistry and reverse transcription polymerase chain reaction. The injection of human placental MSCs through the portal vein using B-ultrasound guidance was superior to the jugular vein approach in a pig model [7]. MSCs from human umbilical cord blood were able to differentiate into hepatocyte-like cells in vivo and partially repair the liver damage caused by D-gal/lipopolysaccharide in mice [13]. Stem cells isolated from menstrual fluid exhibited the potential to revive liver function in mice with two-thirds partial hepatectomy with menstrual stem cell-derived hepatocyte-like cells detected in the recipient liver [14]. Kuo et al. [5] revealed that both MSCs and MSC-derived hepatocytes could engraft into the damaged liver, transdifferentiate into functional hepatocytes, and rescue liver failure in mice. Intravenous transplantation is superior to intrasplenic methods for treating liver failure.

### Paracrine trophic and immunomodulatory factors

We later found that human BMSC transplantation suppressed D-gal-induced cytokine storms and rescued liver failure in pigs within 7 days, mainly through paracrine effects. At this point, BMSC-derived hepatocytes accounted for only ~4.5% of the pig hepatocytes. We identified Delta-like ligand 4 (Dll4) as an important factor that might help the liver to recover [6]. The Dll4/Notch pathway may contribute to the restoration of biliary injury by regulating tubular morphogenesis [15]. Similar experiments by others demonstrated that the rate of accumulation of transplanted MSC-derived hepatocyte mass was low in the recipient liver [16, 17]. These transdifferentiation data appear too low to explain the significant improvement of liver function.

Recently published articles revealed that MSCs alleviate liver failure mainly through trophic and immunomodulatory factors [18, 19]. These factors support hepatocyte function, promote the proliferation of residual hepatocytes, inhibit hepatocyte apoptosis, reverse liver fibrosis, and promote angiogenesis. Furthermore, MSCs exert anti-inflammatory effects on immune cells through soluble cytokines. MSCs suppress the proliferation of peripheral blood mononuclear cells and decrease the secretion of inflammatory cytokines from immune cells. These trophic and immunomodulatory factors have been reviewed by JJ Alm et al. [20]. They create a regenerative microenvironment and reduce inflammatory injury by restraining life-threating cytokine storms and immunocyte infiltration.

Cell-cell contacts with immune cells might also play a role in immunomodulation [3, 21], but the primary mechanism lies in MSC-conditioned medium containing soluble factors rather than cell-cell contacts [22]. Although many trophic and immunomodulatory factors have been characterized from MSC-conditioned medium, much remains unclear regarding its constituents. Thus, the notion of developing a balanced cocktail by combining several key therapeutic molecules instead of intact MSCs appears far-fetched.

### Extracellular vesicles

The latest work revealed that the administration of MSC-derived EVs exerts a similar therapeutic effect as MSC transplantation. MSC-conditioned medium contains both free soluble factors and EVs. The therapeutic effect of MSC-conditioned medium might be a joint effect of both the free soluble factors and EVs. MSC-derived EVs have been studied in animal models for their tissue-protective effects following acute kidney injury, cardiovascular disease, lung injury, liver injury, and cutaneous wound healing [23]. Progress in these animal models may help us understand the mechanism of MSC-derived EVs in liver failure.

EVs are small spherical membrane vesicles derived from the plasma membrane or from multi-vesicular bodies (MVBs). They can be divided into three categories according to their origin and size: exosomes (30–100 nm), microvesicles (100–1000 nm), and apoptotic bodies (500–2000 nm). Exosomes are produced from the inward budding of endosomal compartments called MVBs. Microvesicles bud directly from the plasma membrane and apoptotic bodies are produced during cell apoptosis [24]. Filled with a selection of nucleic acids, lipids, and proteins, EVs are used to exchange information between cells [25]. Almost all cells secrete EVs in response to different stimuli or environmental circumstances.

Several studies have been conducted regarding research of MSC-derived EVs and liver injury (Table 1). MSC-derived exosomes protected the mouse liver against CCl_4_-induced injury, primarily by activating proliferative and regenerative processes rather than by modulating oxidative stress. Exosomes in vitro suppressed hepatocyte apoptosis induced by acetaminophen and H_2_O_2_ by promoting the protein expression of Bcl-xL [26]. Human umbilical cord mesenchymal stem cell (UCMSC)-derived exosomes relieved CCl_4_-induced liver fibrosis by inhibiting epithelial-to-mesenchymal transition and protecting hepatocytes when they were injected directly into the mouse fibrotic liver. Human UCMSC-derived exosomes show similar beneficial effects on the human liver cell line HL7702 in vitro [27]. Glutathione peroxidase 1, which is contained in human UCMSC-derived exosomes, was reported to play a vital role in the recovery of hepatic oxidant injury [28]. Similarly, we observed the anti-apoptotic capacity of menstrual fluid-derived exosomes in acute liver failure [29].

**Table 1:** Translational studies that employed MSC-derived exosomes to treat liver injury

Disease/model	Origin of exosomes	Method/dose	Therapeutic capacity	References
Carbon tetrachloride (CCl4)-induced liver injury mouse model	Human ESC-derived HuES9.E1 MSCs	Intrasplenic injection of 0.4 μg exosomes (in 100 μl PBS)	Elicited hepatoprotective effects against injury primarily by activating of proliferative and regenerative responses	Cheau Yih Tan [26]
CCl4-induced fibrotic liver mouse model	Human umbilical cord-MSCs	Liver injection of 250 μg hucMSC-Ex of 330 μL PBS	Ameliorate liver fibrosis by inhibiting the epithelial-to-mesenchymal transition and protecting hepatocytes	Tingfen Li [27]
CCl4-induced liver failure mouse model	Human umbilical cord MSCs	Tail vein or intragastric administration of 16 mg/kg exosomes	Promoted the recovery of hepatic oxidant injury via the delivery of GPX1	Yongmin Yan [28]
D-GalN/LPS-induced liver failure mouse model	Human menstrual blood-derived stem cells	Tail vein injection of 1 μg/μl MenSC-Ex in PBS (The volume was not mentioned.)	Markedly improved liver function, enhanced survival rates, and inhibited liver cell apoptosis	Lu Chen [29]

Furthermore, MSC-derived EVs possess immunosuppression abilities similar to those of MSCs [30]. MSC-derived EVs could induce Treg cells and anti-inflammatory cytokines and ameliorate the activated immune system [31]. When co-cultured with peripheral blood mononuclear cells, MSC-derived EVs promoted proliferation of the regulatory T-cell population and inhibited T-cell proliferation [32, 33]. Because of their immunosuppressive nature, MSC-derived EVs are being explored in refractory graft-versus-host disease in a patient [32] and in a clinical trial focusing on type I diabetes [34]. EVs may also contribute to inhibiting D-gal-induced life-threatening cytokine storms due to their immunosuppressive abilities.

The exact mechanism behind the therapeutic effect of MSC-derived EVs remains largely obscure and is a topic of intensive investigation. However, MSC-derived EVs are enriched in signaling proteins, including cytokines, chemokines, interleukins, and growth factors [35]. MSCs might play a role in liver regeneration via transfer of exosome cargoes, which include RNA, proteins, lipids, and DNA. This cargo could be transferred to recipient cells through endocytosis, phagocytosis, or fusion with the cell plasma membrane of the recipient cell. Endocytosis is the most common phenomenon for exosome uptake [36]. MSC-derived exosomes moderated myocardium reperfusion damage and reduced infarct size via proteomic complementation [37]. Factors such as interleukin 1β, interleukin 6, transforming growth factor β, and vascular endothelial growth factor have been found in EVs. Some of these factors could regulate immune cells [38]. Additional factors, the ratio of factors in and outside the vesicles, and the function of these factors remain unclear. By horizontally transferring specific mRNA subsets, microvesicles derived from human liver stem cells promote the hepatic regeneration of residual hepatocytes in a hepatectomy model. The adhesion molecules and mRNAs of exosomes contribute to this effect [39]. MSC-derived microvesicles might ameliorate acute tubular injury through the horizontal transfer of mRNA to activate proliferation [40]. MSC-derived EVs could produce energy to support cell survival and convey proteins and RNA to promote angiogenesis and suppress apoptosis [41].

In addition, EVs could regulate target cells through ligand-to-receptor binding. Through binding membrane receptors present mostly on immune cells [42], EVs could modulate the immune response. In conjunction with the ligands for death receptors, EVs could regulate cell death [43]. Both endothelial and tumor cells have been reported to secrete Dll4. Dll4 is a type I transmembrane protein that exerts its function through membrane bound. Endothelial cells can secrete EV-associated Dll4 to alter Notch signaling, which might induce angiogenesis and increase vessel density [44]. The blockade of Dll4 leads to the induction of non-productive angiogenesis [45]. Therefore, we infer that Dll4, which we identified in our previous work [6], might be incorporated into exosomes by MSCs to exert effects in liver regeneration. 

In conclusion, MSC-derived EVs could inhibit the storm of inflammatory factors, suppress apoptosis, promote angiogenesis, provide energy support, promote hepatocyte proliferation, and contribute to biliary restoration to reverse liver failure via cargo transfer and membrane bound [41]. MSC-derived EVs have shown striking therapeutic benefits against liver failure and may be an alternative to MSC therapy. However, the exact mechanism responsible for this activity requires further exploration.

## Conclusions

Our understanding of the role of MSCs in liver failure has continued to develop. Currently, we believe that MSCs can promote liver regeneration by engrafting into damaged liver tissue and transdifferentiating into hepatocytes and by secreting trophic and immunomodulatory factors and EVs (Additional file 1: Figure S1). All these processes may exert effects, but their extent and timing are different. Transdifferentiation might mainly work in the later stage because it takes time for MSCs to transdifferentiate into hepatocytes. Hepatocytes derived from MSCs may have certain compensatory effects in liver failure. It takes approximately 21 days for MSCs to differentiate into hepatocyte-like cells in vitro [4, 7]. The secreted trophic and immunomodulatory factors and EVs can function as soon as MSCs are transplanted. They inhibit hepatocyte apoptosis, reverse liver fibrosis, support hepatocyte function, promote angiogenesis, promote hepatocyte proliferation, contribute to biliary restoration, and reduce inflammatory injury by promptly restraining life-threatening cytokine storms and immunocyte infiltration. Therefore, we infer that MSC-derived factors and EVs primarily work in acute liver failure and that transdifferentiation, EVs, and factors all play therapeutic roles in chronic liver failure. Especially, MSC-derived EVs could exert therapeutic effects independent of whole cells and are promising as a cell-free modality for liver failure treatment.

In liver failure, MSCs are transplanted into unfavorable environments through intravenous, intrahepatic, or intrasplenic methods. MSCs may have difficulty adapting to new environments and may therefore die, transdifferentiate into unwanted myofibroblasts, exert fibrogenic effects, or even undergo malignant transformation [18]. MSCs are not guaranteed to migrate to the injured liver tissue, receive the appropriate signal stimulation to transdifferentiate into hepatocytes, or secrete the most effective factors—although some MSCs may, many may not. Receiving the appropriate stimulatory signal appears to be associated with the time point and route of MSC transplantation [18]; thus, the optimal route, timing, dose, source, and duration should be determined in subsequent experiments, including clinical trials with a long-term follow-up. In addition, promoting the cell growth of pre-existing tumors through MSC injection remains to be clarified.

Using MSC-derived EVs as a surrogate for MSC therapies entails several advantages. EVs are easier to produce in advance, more stable in storage and transportation, and more convenient to control in terms of the quality and quantity. EVs are likely to be less immunogenic, less tumourigenic, and less likely to carry infections such as cytomegalovirus and herpes simplex virus [23]. They are ideal for conveying encapsulated drugs or molecules, which are protected from various degradative enzymes or chemicals in the bloodstream. In particular, EVs could deliver cargoes that operate with stability and integrity into the recipient cell. Previous results in pig models have indicated that intra-portal administration of MSCs was superior to peripheral vein transfusion. This finding might be because many MSCs administered through the peripheral vein were entrapped in the lung [7]. EVs are small vesicles that may bypass the lung when they are administered through the peripheral vein. 

Although two clinical studies associated with MSC-derived EVs have been conducted [32, 34], a series of issues regarding their standardization, large scale production, quality control, optimal dosing, biodistribution, and safety should be addressed before they come into clinical use in liver failure. Using EVs for clinical treatment requires large quantities of EVs, so increasing the production and simplifying production process are necessary. Preconditioning or genetic modification of MSCs could be utilized to increase the production and quality of EVs. Modifying EVs could also be explored. Intracellular calcium levels, external stress, and cytoskeletal fixation are being explored to remove this obstacle [24]. In addition, MSC-derived EVs might contribute to the growth and aggressiveness of tumors [46]. There are several subtypes of EVs, and we should identify and purify EVs by eliminating those vesicles or molecules that might be harmful to the body. Furthermore, we could use engineering techniques to knock out harmful molecules, produce and enrich effective molecules in MSC-derived EVs, and deliver those EVs accurately to recipient cells to treat liver failure.

## Additional file

Additional file 1: Figure S1. Transdifferentiation and paracrine effects of MSCs in liver failure. MSCs could migrate to the damaged tissue, transdifferentiate into hepatocytes, and replace the damaged cells. Furthermore, MSCs could exert trophic and immunomodulatory effects by secreting cytokines and EVs. EVs function through cell surface membranes and cargoes transfer by fusion with the plasma membrane or endocytosis. These EVs contain various molecules, including RNA, DNA, lipids, and proteins. (JPG 168 kb)

## Abbreviations

BMSC: Bone marrow-derived mesenchymal stem cell
D-gal: D-galactosamine
Dll4: Delta-like ligand 4
EV: Extracellular vesicle
MSC: mesenchymal stem cell
MVB: Multi-vesicular body
UCMSC: umbilical cord mesenchymal stem cell.
