# Supplementary figures and images for: Role of mesenchymal stem cells, their derived factors, and extracellular vesicles in liver failure
Source: Stem Cell Res Ther. 2017 Jun 6;8:137. doi: 10.1186/s13287-017-0576-4 (PMC5460333; doi:10.1186/s13287-017-0576-4)

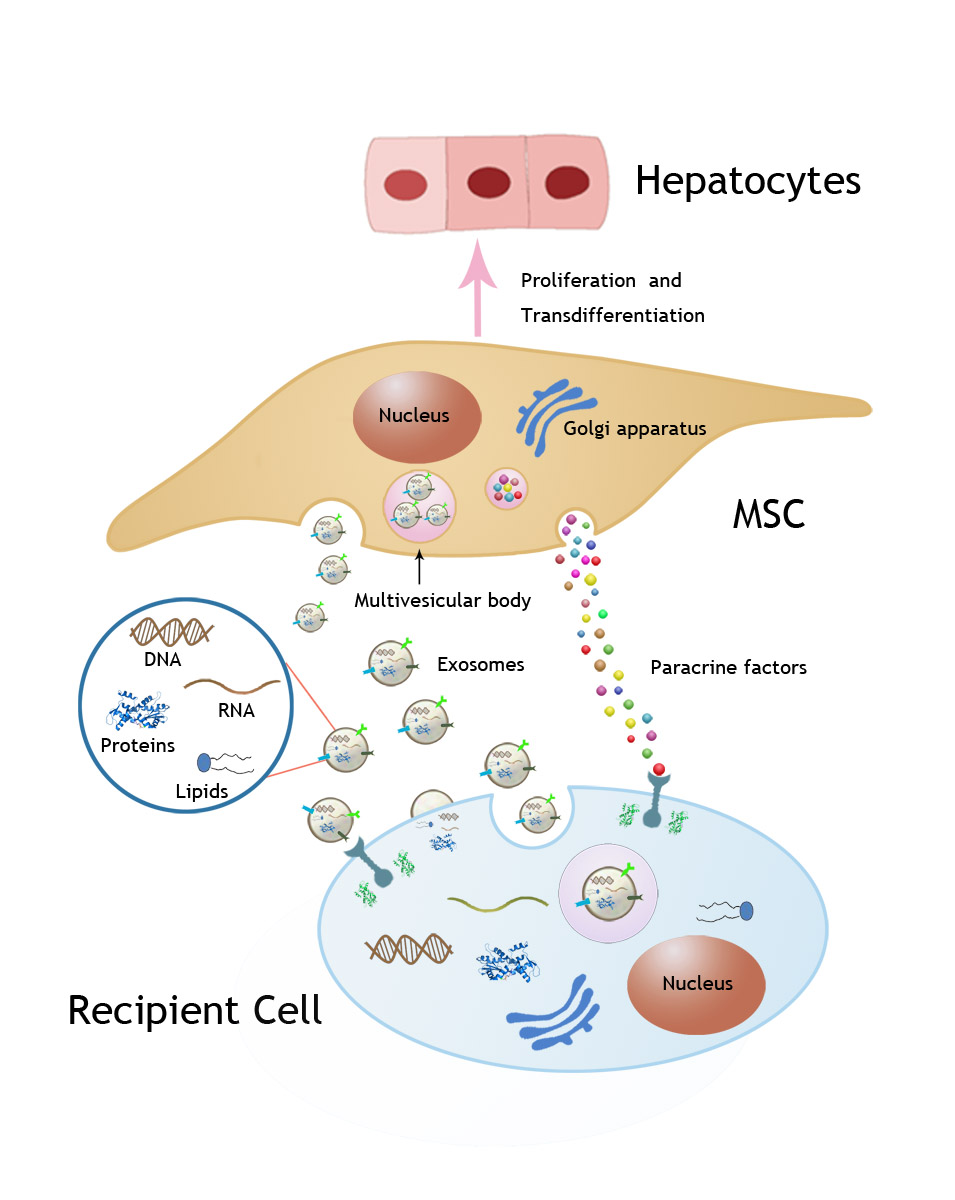

Supplement: Additional file 1: Figure S1. — Transdifferentiation and paracrine effects of MSCs in liver failure. MSCs could migrate to the damaged tissue, transdifferentiate into hepatocytes, and replace the damaged cells. Furthermore, MSCs could exert trophic and immunomodulatory effects by secreting cytokines and EVs. EVs function through cell surface membranes and cargoes transfer by fusion with the plasma membrane or endocytosis. These EVs contain various molecules, including RNA, DNA, lipids, and proteins. (JPG 168 kb) [file 13287_2017_576_MOESM1_ESM.jpg]
